# Therapeutic targeting of vascular malformation in a zebrafish model of hereditary haemorrhagic telangiectasia

**DOI:** 10.1242/dmm.049567

**Published:** 2023-03-30

**Authors:** Ryan O. Snodgrass, Karan Govindpani, Karen Plant, Elisabeth C. Kugler, Changmin Doh, Thomas Dawson, Luis E. McCormack, Helen M. Arthur, Timothy J. A. Chico

**Affiliations:** ^1^ Department of Infection, Immunity and Cardiovascular Disease, Medical School, University of Sheffield, Sheffield S10 2RX, UK; ^2^ Biosciences Institute, Centre for Life, Newcastle University, Newcastle NE1 3BZ, UK

**Keywords:** Zebrafish, Endoglin, Angiogenesis, HHT, VEGF

## Abstract

Hereditary haemorrhagic telangiectasia (HHT) causes arteriovenous malformations (AVMs) in multiple organs to cause bleeding, neurological and other complications. HHT is caused by mutations in the BMP co-receptor endoglin. We characterised a range of vascular phenotypes in embryonic and adult *endoglin* mutant zebrafish and the effect of inhibiting different pathways downstream of Vegf signalling. Adult *endoglin* mutant zebrafish developed skin AVMs, retinal vascular abnormalities and cardiac enlargement. Embryonic *endoglin* mutants developed an enlarged basilar artery (similar to the previously described enlarged aorta and cardinal vein) and larger numbers of endothelial membrane cysts (kugeln) on cerebral vessels. Vegf inhibition prevented these embryonic phenotypes, leading us to investigate specific Vegf signalling pathways. Inhibiting mTOR or MEK pathways prevented abnormal trunk and cerebral vasculature phenotypes, whereas inhibiting Nos or Mapk pathways had no effect. Combined subtherapeutic mTOR and MEK inhibition prevented vascular abnormalities, confirming synergy between these pathways in HHT. These results indicate that the HHT-like phenotype in zebrafish *endoglin* mutants can be mitigated through modulation of Vegf signalling. Combined low-dose MEK and mTOR pathway inhibition could represent a novel therapeutic strategy in HHT.

## Teaser

**Summary:** The vascular abnormalities in a zebrafish genetic model of hereditary haemorrhagic telangiectasia were prevented by synergistic inhibition of the MEK and mTOR pathways downstream of Vegf, suggesting a potential novel therapeutic approach.

## INTRODUCTION

Endoglin (ENG) is highly expressed in endothelial cells (ECs), and regulates the development and maintenance of blood vessels (Arthur et al., 2000; Jin et al., 2017; Mahmoud et al., 2010). ENG is a high-affinity co-receptor for BMP9 and BMP10 ligands of the TGF-β superfamily (Castonguay et al., 2011; Scharpfenecker et al., 2007). It serves as a BMP9/10 ligand reservoir on the EC surface (Lawera et al., 2019) and promotes ligand-induced ACVRL1 phosphorylation of SMAD1/5/8 (Lebrin et al., 2004). Once phosphorylated, these transcription factors bind to SMAD4 to modulate expression of genes regulating angiogenesis and vascular morphology. Reduced ENG or ACVRL1 activity, owing to loss-of-function mutations, leads to the human disease hereditary haemorrhagic telangiectasia (HHT) (Johnson et al., 1996; McAllister et al., 1994; Snodgrass et al., 2021). HHT is characterised by arteriovenous malformations (AVMs). Large AVMs may affect the lungs, liver and brain, while telangiectases arise from smaller AVMs between a postcapillary venule and arteriole in mucocutaneous tissues and are prone to rupture and haemorrhage (Guttmacher et al., 1995; Shovlin, 2010).

Murine studies (Tual-Chalot et al., 2015) show that *Eng*-null mice die by gestational day 11.5 due to defective blood vessel and heart development (Arthur et al., 2000; Li et al., 1999). Loss of endothelial ENG in neonatal mice leads to retinal AVMs. These result from increased endothelial proliferation and defective EC migration against blood flow during angiogenesis (Jin et al., 2017; Mahmoud et al., 2010). A similar AVM phenotype is seen in neonatal retinas of mice without endothelial ACVRL1 due to uncoupling of haemodynamic forces with EC migration (Tual-Chalot et al., 2014; Park et al., 2021). Loss of endothelial ENG in adult mice causes peripheral AVMs associated with increased vessel growth, leading to high-output heart failure. Furthermore, AVMs caused by endothelial loss of ENG can be corrected by overexpression of ACVRL1 (Hwan Kim et al., 2020), confirming that ENG is the upstream co-receptor for BMP9/10 in the ENG/ACVRL1/SMAD1,5,8 signalling pathway.

In zebrafish embryos, loss of *eng* leads to arteriovenous shunting between an enlarged dorsal aorta (DA) and an enlarged posterior cardinal vein (PCV) (Sugden et al., 2017). These vessels show increased EC size and increased blood flow, with reduced flow through the intersegmental vessels (Sugden et al., 2017). These defects reflect reduced sensitivity to haemodynamic cues following loss of ENG (Baeyens et al., 2016).

ENG thus mediates multiple EC responses, including proliferation, migration against flow and cellular footprint in the context of angiogenic cues such as VEGF. As ENG promotes BMP9/10 signalling through ACVRL1, further insights into the importance of this pathway in vascular development and remodelling come from *acvrl1* and *bmp10* zebrafish mutants. Loss of *acvrl1* function leads to increased basilar artery (BA) diameter and AVMs in the embryonic cranial vasculature. This is due to accumulation of ECs caused by reduced migration of ECs (Corti et al., 2011). *bmp10* mutant zebrafish also develop enlarged cranial vessels and high cardiac output associated with dermal and hepatic vascular defects (Capasso et al., 2020). ENG promotes BMP9/BMP10/ACVRL1 signalling to couple haemodynamic cues to enable EC migration during development and remodelling of the vasculature (Roman and Hinck, 2017). Disrupting this pathway leads to AVMs (Roman and Hinck, 2017). However, the underlying molecular mechanisms leading to AVM formation in HHT are still not fully understood, and a better understanding of these is required to improve treatment.

Targeting VEGF signalling may ameliorate HHT. ENG interacts with the VEGF receptor VEGFR2 (KDR) (Tian et al., 2018), and loss of ENG leads to increased activation of VEGFR2 in response to VEGF ligand (Arthur and Roman, 2022; Tual-Chalot et al., 2020). This suggests that the phenotype of ENG loss of function results from increased VEGF signalling. Supporting this, the anti-VEGF inhibitor bevacizumab reduces epistaxis and high-output heart failure in HHT patients with hepatic AVMs (Dupuis-Girod et al., 2012). Furthermore, blocking VEGF signalling reduces vascular malformations in mouse models of HHT1 and HHT2 (Han et al., 2014; Tual-Chalot et al., 2020). However, serious adverse cardiovascular complications can be caused by inhibiting VEGF directly (Totzeck et al., 2017). VEGF signalling is complex and drives numerous downstream pathways (Fig. S1). Targeting specific downstream pathways of VEGF may provide benefit with reduced risk of side-effects.

To address which pathway (or combination of pathways) could provide therapeutic effects in HHT, we took advantage of the zebrafish embryo's suitability for pharmacological assays, including rapid cardiovascular development, ease of drug administration and transgenic tools for imaging. The *Tg(kdrl:Hsa.HRAS-mCherry)^s916^* (Chi et al., 2008) transgenic reporter labels the EC membrane, facilitating live imaging of the embryonic trunk and cranial vasculature, and imaging of vasculature in explanted tissue. We previously used this reporter to describe a novel EC behaviour, in which the cerebral vessels form structures termed kugeln – transient spherical protrusions that extrude abluminally from cerebral blood vessels. Unlike other membrane protrusions, kugeln are neither driven by blood flow nor contain cytoplasm (Kugler et al., 2019). Kugeln are only present on cerebral vessels, and although their function is unclear, they may provide new insights into mechanisms of cerebrovascular development and function. Here, in addition to the previously reported enlargement of major trunk vessels in *eng* mutant zebrafish embryos, we discovered an increase in kugel formation, as well as increased BA diameter, in *eng* mutants. We pharmacologically inhibited either global Vegf signalling or components of different pathways downstream of VEGFR2 (Kdrl) (Fig. S1) in zebrafish *eng* mutant and control embryos, and identified MEK/ERK (MAP3K/MAPK) and mTOR as synergistic therapeutic targets in HHT.

## RESULTS

### Adult *eng* mutant zebrafish display cardiovascular defects

**Fig. 1.**
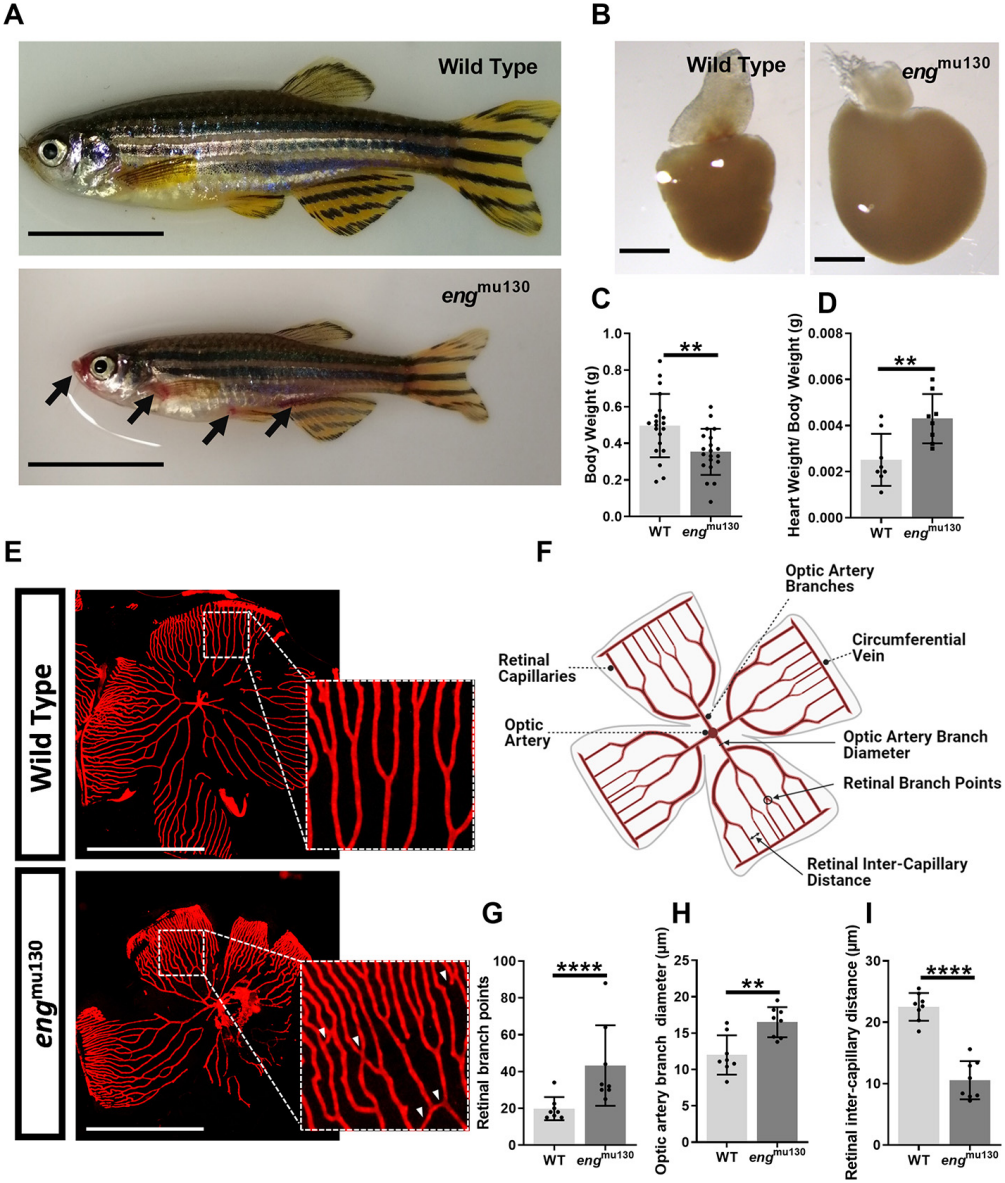
**Adult *eng*^mu130^ fish display cutaneous and retinal vascular malformations similar to those in patients with hereditary haemorrhagic telangiectasia and an enlarged heart.** (A) Adult 5- to 6-month-old *eng*^mu130^ zebrafish display cutaneous vascular malformations (black arrows). Scale bars: 1 cm. (B) Representative *eng*^mu130^ and wild-type (WT) hearts (*n*=5/group). Scale bars: 500 μm. (C) Body weight of adult WT and *eng*^mu130^ zebrafish. (D) Cardiac ventricle weight/body weight of WT and *eng*^mu130^ zebrafish. (E) Fluorescent micrographs of whole-mount retinae from *Tg*(*kdrl:Hsa.HRAS-mCherry*)^s916^ zebrafish, showing abnormal vessel communications in *eng*^mu130^ (white arrowheads). Scale bars: 500 μm. (F) Schematic diagram of a 5- to 6-month-old zebrafish retinal vasculature. Figure created with BioRender. (G) Number of retinal vascular branches in WT and *eng*^mu130^ homozygous siblings. (H) Optic artery diameter in WT and *eng*^mu130^ homozygous siblings. (I) Inter-capillary distance in WT and *eng*^mu130^ homozygous siblings. **P<0.01, ****P<0.0001 (unpaired two-tailed Student's *t*-test, 8-10 animals/group).

Given the prominent cutaneous vascular phenotype of human HHT, we examined *eng* mutants for similar skin phenotypes. We observed that 71% (5/7) of adult *eng*^mu130^ zebrafish displayed large blood-filled arteriovenous malformations, which were not present in wild types (WTs) (0/5) (Fig. 1A).

Although *eng*^mu130^ embryos were similar in size to WT siblings (Fig. S2A), adult *eng*^mu130^ mutant zebrafish adults were smaller and weighed less than WT siblings (Fig. 1A,C). However, hearts were significantly larger in *eng* mutants than in WTs (Fig. 1B,D). This is similar to the phenotype of endothelial-specific *Eng* knockout adult mice (Tual-Chalot et al., 2020).

We next examined the effect of *eng* mutation on retinal vascular patterning in adult zebrafish. Representative micrographs are shown in Fig. 1E and a schematic of the parameters is in Fig. 1F. We found that homozygous *eng* mutation increased vessel branchpoint number (Fig. 1G), increased optic artery branch diameter (Fig. 1H) and reduced inter-capillary distance (Fig. 1I) compared with WT.

Despite these persistent cardiac and vascular abnormalities, homozygous *eng*^mu130^ mutant adults were fertile.

### Embryonic *eng* mutant zebrafish display cardiovascular defects

In normal zebrafish development, the DA and PCV run along the axis of the trunk, connected by the dorsal longitudinal anastomotic vessel (DLAV) (Fig. S3A). The diameter of the DA and PCV reduces between 52 and 100 h post fertilisation (hpf) in WT but not *eng*^mu130^ mutants (Sugden et al., 2017). In agreement with this, we found that the DA and PCV diameters were significantly larger in *eng*^mu130^ mutants than in WTs, and were most pronounced by 100 hpf (Fig. S3B-D). Increased flow through the enlarged major vessels in *eng^mu130^* mutants is associated with reduced flow in intersegmental blood vessels (ISVs) and delayed opening of ISV lumens (Sugden et al., 2017), a finding we also confirm (Fig. S3E,F). As previously shown, this is due to an increase in EC size and not to increased EC number (Sugden et al., 2017) (Fig. S4A-D).

**Fig. 2.**
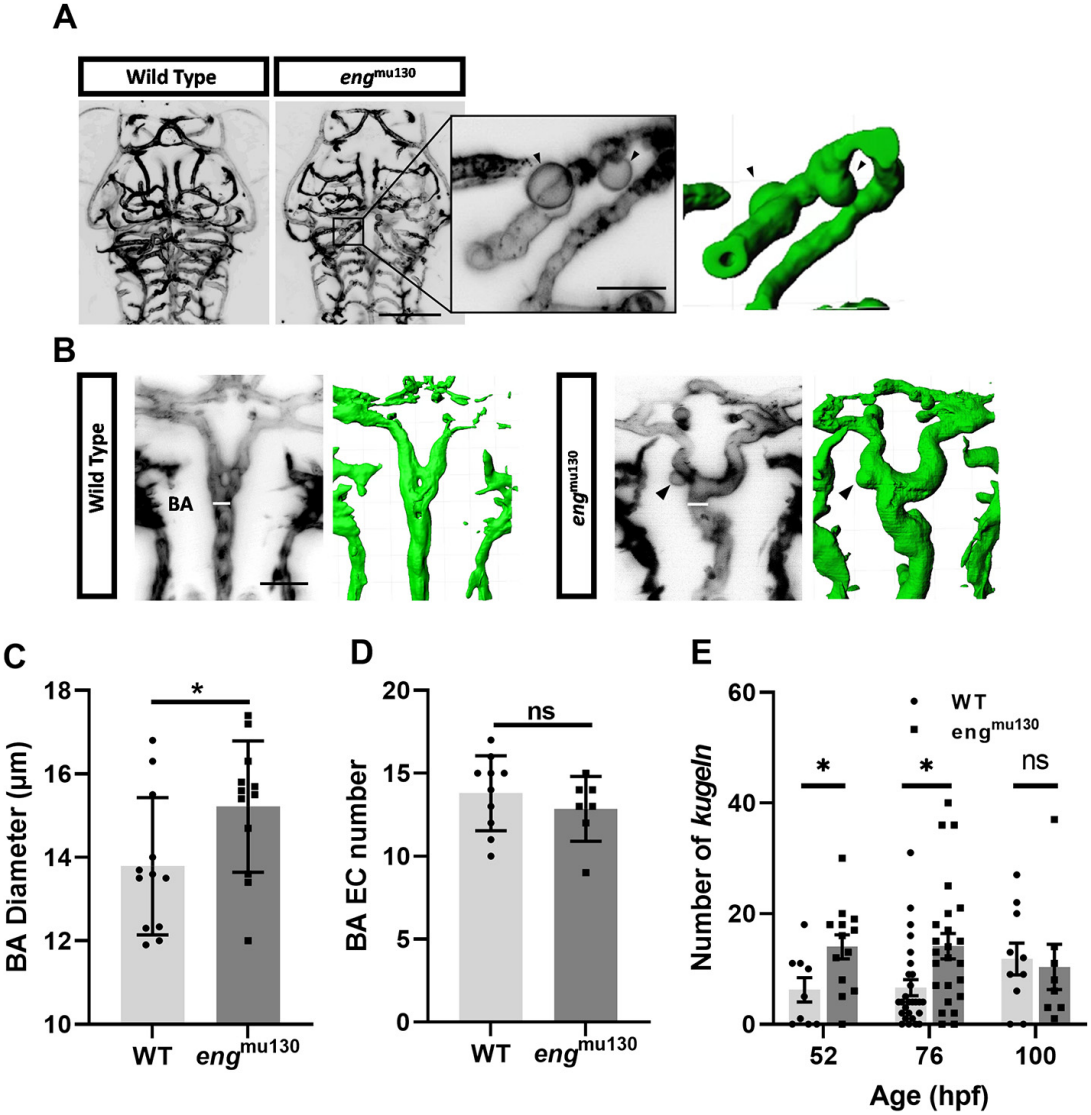
***eng*^mu130^ mutant embryos have increased basilar artery diameter and increased number of endothelial kugeln.** (A) Representative maximum-intensity projection of *Tg(kdrl:Hsa.HRAS-mCherry)^s916^* zebrafish brain vasculature at 72 hpf. Black arrowheads indicate individual kugeln. Scale bars: 150 μm/50 μm (inset). Three-dimensional reconstruction of the same image shown in green is on the right. (B) Representative maximum-intensity projection of the basilar artery (BA) at 72 hpf. Black arrowheads indicate individual kugeln. White bars indicate where diameter of the BA was measured. Scale bar: 50 μm. Three-dimensional reconstructions of the same images shown in green are on the right. (C) BA diameter in *eng*^mu130^ mutants and WT siblings (unpaired two-tailed Student's *t*-test, 10-12 animals/group). (D) *eng* mutation did not alter endothelial cell (EC) number in the BA (unpaired two-tailed Student's *t*-test, 10-12 animals/group). (E) Kugel number per animal was increased in *eng*^mu130^ mutants at 52 and 74 h post fertilisation (hpf) but not at 100 hpf. *P<0.05; ns, not significant (Mann–Whitney *U*-test, 8-27 animals/group).

To determine whether developmental abnormalities induced by *eng* mutation were seen in additional vascular beds, we examined the zebrafish cranial vasculature at 52-100 hpf. We observed that *eng*^mu130^ mutants developed significantly more cerebral vessel kugeln than did WT siblings at 52 and 74 hpf but not at 100 hpf (Fig. 2A,E). The BA had an increased diameter, but no increase in EC number, in *eng*^mu130^ mutants compared with WT siblings (Fig. 2B-D). This is similar to the enlarged BA in *acvrl1* and *bmp10* mutant zebrafish (Capasso et al., 2020; Corti et al., 2011). We used a recently established automated analysis pipeline (Kugler et al., 2022) to quantify a range of other parameters of cerebrovascular patterning and found that the *eng* mutation did not affect central artery number, posterior midbrain channel width, total branch point number, mean vessel radius or total network length compared with WT at 72 hpf (Fig. S5).

Thus, we find a range of transient and persistent abnormal vascular phenotypes in zebrafish *eng* mutants.

### Abnormal brain and trunk vasculature of *eng^mu130^* zebrafish embryos is prevented by VEGFR2 inhibition

**Fig. 3.**
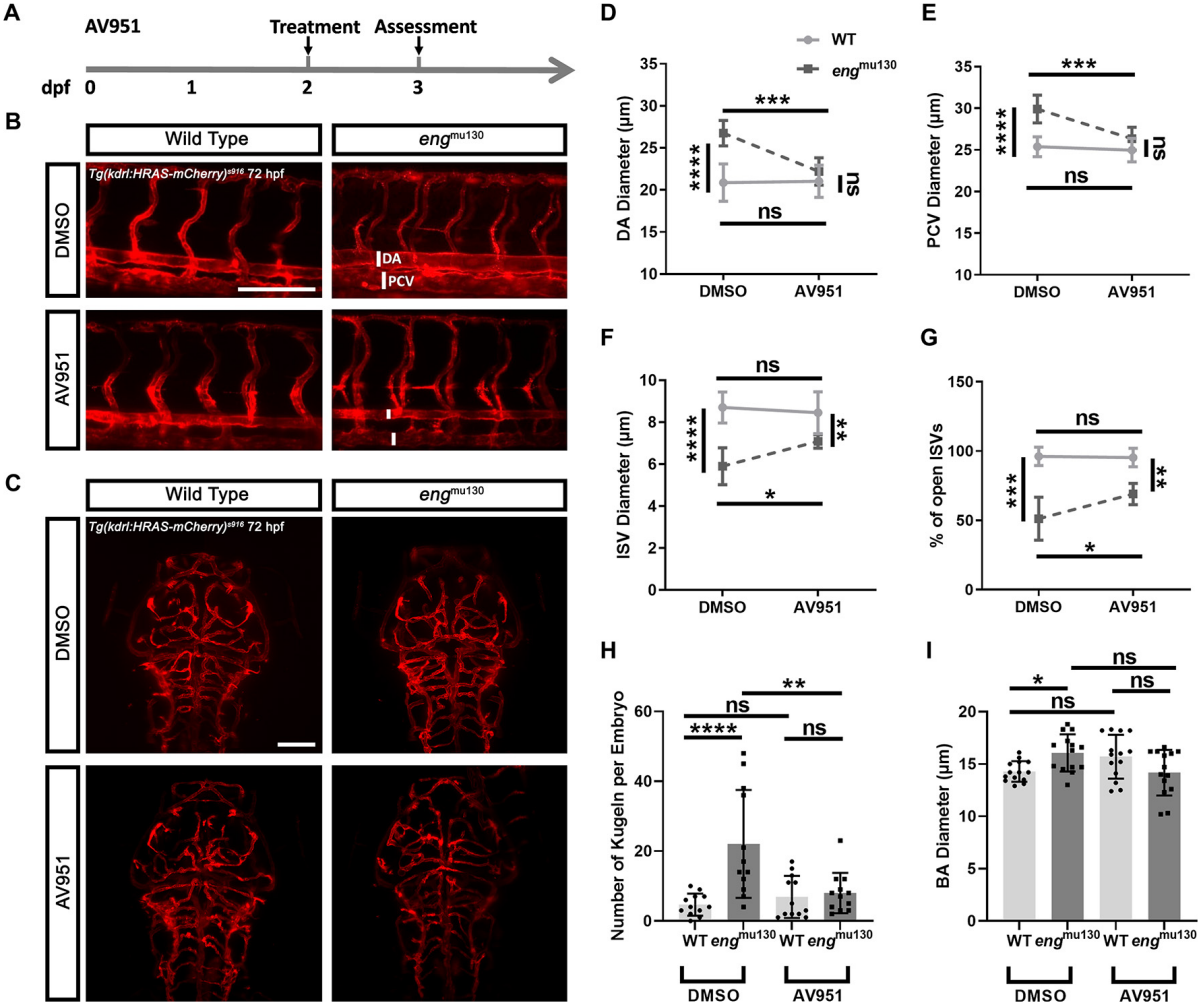
**Vegf inhibition between 2 and 3** **dpf rescues the abnormal trunk and cerebral vessel phenotypes of *eng*^mu130^ mutants.** (A) Experimental plan for rescuing the *eng* mutant phenotype in zebrafish using AV951. dpf, days post fertilisation. (B) Representative maximum-intensity projection of the trunk vasculature of *eng*^mu130^ and WT embryos±AV951 (25 nM) treatment for 24 h. DA, dorsal aorta; PCV, posterior cardinal vein. Scale bar: 150 μm. (C) Representative maximum-intensity projection of the cerebral vasculature of *eng*^mu130^ and WT embryos±AV951 (25 nM) treatment for 24 h. Scale bar: 100 μm. (D) DA diameter in *eng*^mu130^ and WT embryos±AV951 treatment. (E) PCV diameter in *eng*^mu130^ and WT embryos±AV951 treatment. (F) Intersegmental blood vessel (ISV) diameters in *eng*^mu130^ and WT embryos±AV951 treatment. (G) Percentage of open ISVs in *eng*^mu130^ and WT embryos±AV951 treatment. (H) Number of kugeln in *eng*^mu130^ and WT embryos±AV951 treatment. (I) BA diameter in *eng*^mu130^ and WT embryos±25 nM AV951 treatment. *P<0.05, **P<0.01, ***P<0.001, ****P<0.0001; ns, not significant (two-way ANOVA with Tukey post-hoc test, 10 animals/group).

As loss of ENG disrupts integration of BMP9/10 and VEGF signalling pathways, we investigated the effect of inhibiting Vegf signalling in *eng*^mu130^ zebrafish. Treating *eng*^mu130^ embryos between 2 and 3 days post fertilisation (dpf) with the VEGFR2 receptor tyrosine kinase inhibitor AV951 (tivozanib) prevented enlargement of the DA and PCV in *eng* mutants (Fig. 3A,B,D,E). AV951 treatment between 2 and 3 dpf also normalised ISV diameter and perfusion (Fig. 3A,B,F,G). These effects were not due to general toxicity or non-specific effects on vascular development, as the same treatment did not affect heart rate (Fig. S2B), DA diameter, PCV diameter, ISV diameter or perfusion in WT animals (Fig. 3B,D-G). This indicates that the HHT-like phenotype in zebrafish *eng* mutants can be mitigated by Vegf inhibition.

We next asked whether the cerebral vessel phenotype of *eng* mutants is caused by a similar dysregulation of Vegf signalling. Treating *eng*^mu130^ embryos between 2 and 3 dpf with AV951 did not affect cerebral vascular patterning (Fig. 3C). However, similarly to its effect on the trunk vasculature, AV951 prevented increased kugel formation in the mutant embryos at 72 hpf, without affecting kugel number in WT siblings (Fig. 3H). The increased BA diameter in *eng* mutants was also prevented by treatment with AV951, whereas the same treatment did not alter BA diameter in WTs (Fig. 3I). Because there is already a detectible vascular phenotype in *eng* mutants at 2 dpf, these findings suggest that Vegf inhibition can reverse, as well as prevent, abnormal vascular phenotypes in *eng* mutants.

### mTOR and MEK inhibition prevents abnormal brain and trunk vasculature of *eng^mu130^* zebrafish embryos

**Fig. 4.**
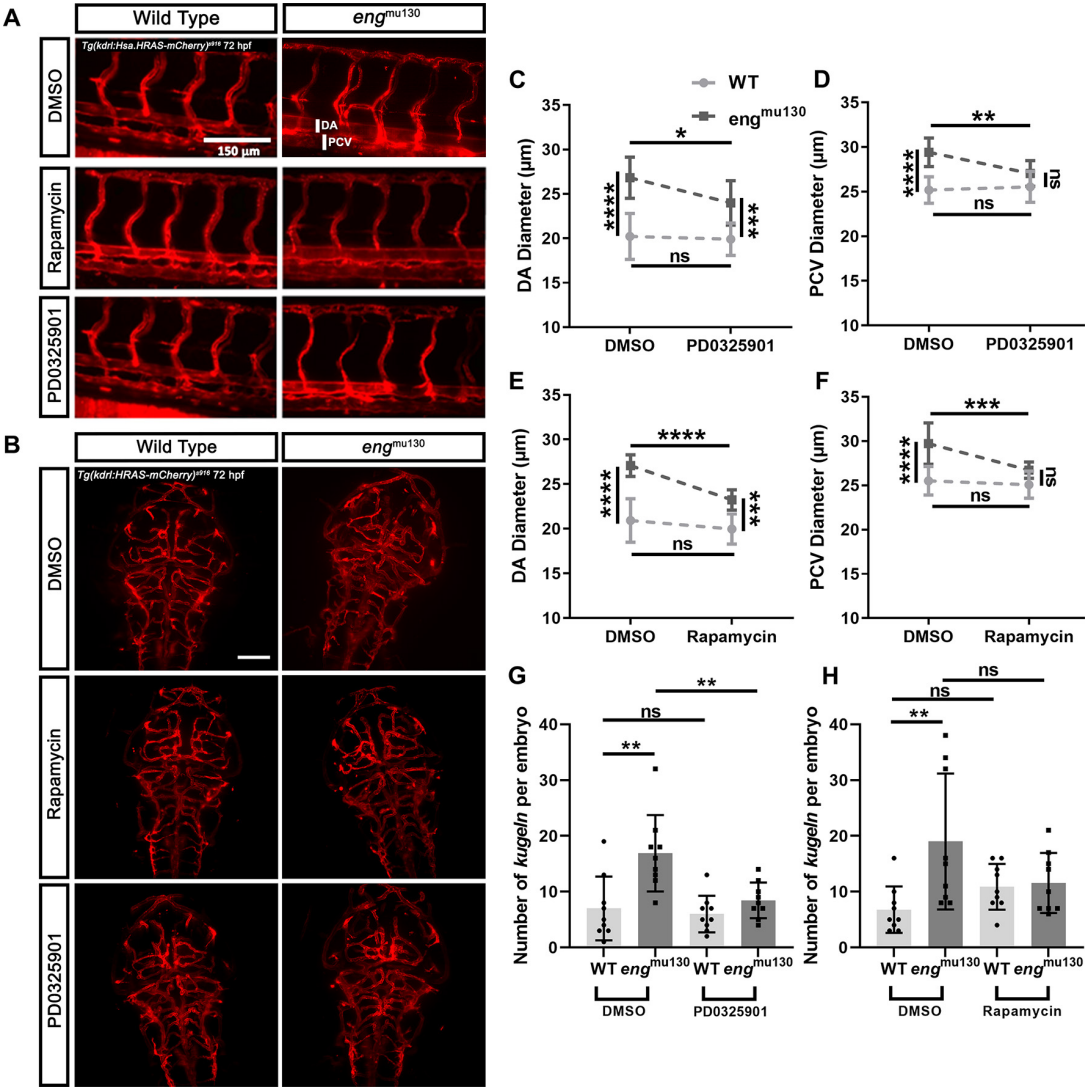
**MEK or TOR inhibition normalises the vascular phenotype of *eng*^mu130^ embryos.** WT and *eng*^mu130^ mutant embryos were treated with the MEK inhibitor PD0325901 (10 µM) or with the TOR inhibitor rapamycin (2.5 µM) for 24 h between 2 and 3 dpf, or with DMSO vehicle. (A) Representative maximum-intensity projections of the trunk vessels of *eng*^mu130^ and WT embryos±PD0325901 (10 µM) or rapamycin (2.5 µM) treatment for 24 h. Scale bar: 150 μm. (B) Representative maximum-intensity projections of the cerebral vessels of *eng*^mu130^ and WT embryos±PD0325901 (10 µM) or rapamycin (2.5 µM) treatment for 24 h. Scale bar: 100 μm. (C,D) *eng*^mu130^ mutant embryos show reduced DA (C) and PCV (D) diameter following PD0325901 treatment, with PCV diameter normalised to WT values. (E,F) *eng*^mu130^ mutant embryos show reduced DA diameter (E) and PCV diameter (F) following rapamycin treatment, with PCV diameter normalised to WT values. (G) *eng*^mu130^ mutant embryos show normalised kugel formation following PD0325901 treatment. (H) *eng*^mu130^ mutant embryos show normalised kugel formation following rapamycin treatment. *P<0.05, **P<0.01, ***P<0.001, ****P<0.0001; ns, not significant (two-way ANOVA with Tukey post-hoc test, 10-14 animals/group).

VEGFR2 signalling is complex and signals downstream via several pathways. *In vitro*, BMP9 decreases VEGF signalling, and loss of BMP9 signalling through ACVRL1 or ENG depletion leads to increased phosphorylated (p)ERK levels (Alsina-Sanchís et al., 2018; Tual-Chalot et al., 2020). We treated 2 dpf embryos for 24 h with 10 µM PD0325901, a MEK inhibitor. Fig. 4A shows trunk vasculature of representative WT and *eng* mutants treated with the inhibitor or vehicle. MEK inhibition prevented the enlarged DA and PCV of *eng*^mu130^ embryos but did not affect the axial vessel diameter of WT siblings (Fig. 4C,D).

When we examined the cerebral vasculature, MEK inhibition did not alter cerebral vascular patterning in either WT or *eng*^mu130^ embryos (Fig. 4B). However, MEK inhibition did reduce the number of kugeln to WT levels (without affecting kugel number in WTs) (Fig. 4G).

Another major pathway downstream of VEGFR2 is the PI3K/AKT pathway, which affects both mTOR and eNOS (NOS3) function to regulate cell survival and vasoregulation, respectively. Targeting PI3K can reduce AVMs in murine HHT2 models (Ola et al., 2016). Furthermore, in a model of HHT caused by loss of BMP9 and BMP10 ligands, endothelial mTOR levels are increased and targeting mTOR reduces vascular defects (Ruiz et al., 2020). Therefore, we assessed the effect of the mTOR inhibitor rapamycin on *eng*^mu130^ zebrafish embryos. We found that rapamycin had very similar effects to MEK inhibition. Specifically, it prevented the *eng*^mu130^ phenotypes for DA, PCV, ISVs, BA and kugel number without affecting WT embryos (Fig. 4A,B,E,F,H).

Next, we examined the effect of MEK and mTOR inhibition on the cerebral vascular phenotype of *eng* mutants. PD0325901 (10 µM) prevented the increase in number of kugeln in *eng*^mu130^ embryos, but did not affect kugel number in WT animals (Fig. 4G). This suggests that upregulation of MEK/ERK signalling due to *eng* loss of function contributes to the trunk vessel abnormalities and increased kugel formation in *eng* mutants. Rapamycin (2.5 µM) similarly prevented the increase in kugel number in *eng* mutant embryos without altering kugel number in WTs (Fig. 4H).

eNOS is a critical enzyme regulating levels of nitric oxide (NO) upregulated both by flow and VEGF signalling, and there is evidence that ENG regulates coupling of eNOS activity (Toporsian et al., 2005). However, we found no detectable effect on DA or PCV vessel size in *eng*^mu130^ embryos following treatment with the NOS inhibitor L-NAME (Fig. S6A,B). Furthermore, treatment of embryos at 2 dpf for 24 h with 10 µM SB203580 to inhibit p38 MAPK (Mapk14) did not affect DA and PCV vessel size in *eng*^mu130^ embryos (Fig. S6C,D).

**Fig. 5.**
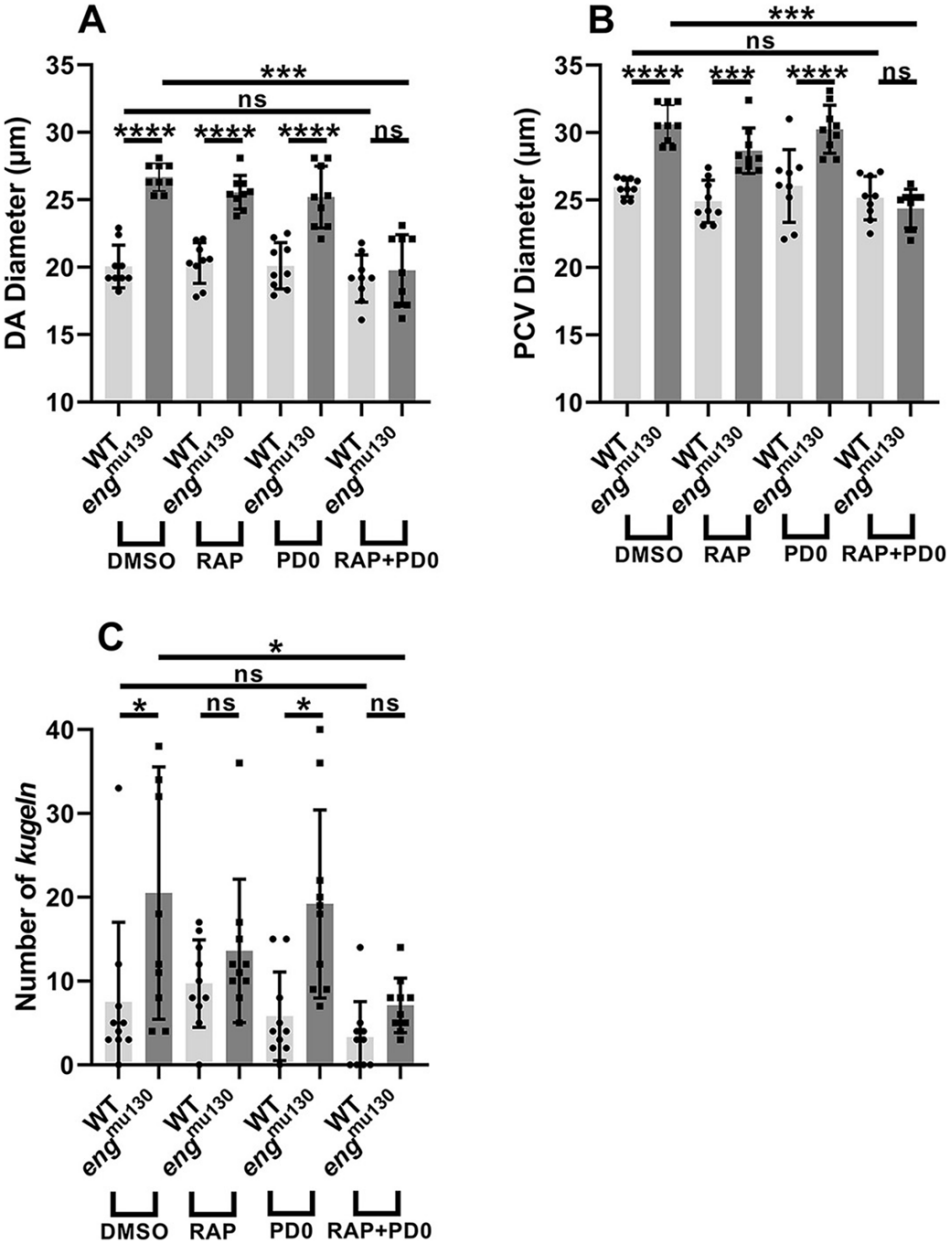
**Combined low-dose mTOR and MEK inhibition prevents abnormal trunk vasculature and excess kugel formation in *eng*^mu130^ zebrafish embryos.** (A-C) Neither 2 µM rapamycin (RAP) nor 7.5 µM PD0325901 (PD0) alters the vascular phenotype of *eng*^mu130^ mutants, but combined treatment resulted in normalised DA and PCV diameters, as well as reduced kugel formation in *eng*^mu130^ zebrafish embryos. *P<0.05, ***P<0.001, ****P<0.0001; ns, not significant (two-way ANOVA with Tukey post-hoc test, 10-14 animals/group).

Finally, we investigated whether combined low-dose mTOR and MEK inhibition prevents abnormal trunk vasculature in *eng*^mu130^ embryos. Neither 2 µM rapamycin nor 7.5 µM PD0325901 alone altered the vascular phenotype of *eng*^mu130^ mutants, but co-treatment normalised both DA and PCV diameters and reduced the excess kugel formation phenotype (Fig. 5A-C), suggesting that the MEK/ERK and Akt/mTOR pathways jointly and syngergistically contribute to the abnormal vessel phenotype in *eng* mutants.

## DISCUSSION

Our findings substantially expand the previously described vascular phenotype in *eng*^mu130^ mutant zebrafish. Adult *eng* mutants display cutaneous vascular lesions similar to those of HHT patients, as well as retinal vascular abnormalities and cardiac enlargement. In *eng* mutant embryos, we uncover a striking cerebrovascular phenotype of enlarged BA and increased endothelial kugel formation. Currently, the physiological significance of kugeln is not clear, but this is the first work that implicates kugel formation in a model of human disease.

Treatment of *eng*^mu130^ embryos with AV951 to target VEGFR2 prevented development of the abnormally enlarged major vessels and normalised cerebrovascular kugel number, but had no effect on WT siblings at the concentrations and timepoints assessed. This indicates that the HHT-like phenotype in zebrafish *eng* mutants is likely to relate to increased VEGF-stimulated VEGFR2 phosphorylation and altered VEGFR2 kinetics in ECs (Tual-Chalot et al., 2020; Jin et al., 2017). This is consistent with our results showing that the abnormal phenotype in *eng* mutants can be abrogated by inhibiting Vegf signalling, in agreement with other studies in mouse models of HHT (Han et al., 2014; Jin et al., 2017; Tual-Chalot et al., 2020) and current clinical therapies targeting VEGF in HHT patients (Dupuis-Girod et al., 2012). However, anti-VEGF treatment has to be continuous or semi-continuous to maintain suppression of abnormal angiogenic responses, and anti-VEGF therapies such as bevacizumab have severe side-effects and require repeated intravenous delivery (Totzeck et al., 2017). VEGF signalling activates numerous downstream pathways (Fig. S1); knowing which of these is responsible for the phenotypes in HHT will help to guide more focused therapeutic interventions.

We therefore took advantage of the zebrafish embryo's suitability for drug-screening assays, including its rapid cardiovascular development, *ex utero* fertilisation and rapid uptake of inhibitors from the incubating medium to target separate signalling pathways downstream of VEGFR2 in *eng*^mu130^ embryos. As mouse *Eng* mutants show increased pERK activity in ECs downstream of VEGF signalling, and increased pERK was observed in ECs associated with AVMs (Tual-Chalot et al., 2020), this may represent a key pathway to target downstream of VEGF. Indeed, sporadic human cerebral AVMs also have an exaggerated pERK response, suggesting that aberrant pERK activation is a common feature of AVMs (Jakobsson and Arthur, 2020; Nikolaev et al., 2018). Treatment of embryos for 24 h between 2 and 3 dpf with PD0325901 to inhibit MEK, upstream of ERK, normalised the enlarged vessels and reduced the excess kugel formation in *eng*^mu130^ zebrafish embryos. These timings were chosen as pERK is present at an early stage (from 18 hpf) in the developing DA, and predefines ECs that are going to contribute to ISV formation (Nagasawa-Masuda and Terai, 2016). However, this initial process is completed by 2 dpf when MEK inhibition was initiated. Importantly, development and vessel size (including ISV) of WT embryos was unaffected by exposure to PD0325901. Thus, targeting MEK effectively prevented occurrence of the *eng* mutant vascular phenotype. In contrast, targeting p38 MAPK, a second pathway downstream of VEGF signalling, had no detectable effect on the enlarged trunk vessels in *eng* mutants, suggesting that altered p38-regulated cell migration was not involved in the HHT phenotype. Finally, we looked at the PI3K/AKT pathway downstream of VEGF. Inhibition of PI3K in *Acvrl1* mouse neonates has previously been shown to protect against development of vascular abnormalities (Alsina-Sanchís et al., 2018; Ola et al., 2016). *Acvrl1*-deficient endothelial cells show enhanced activation of the PI3K/AKT pathway (Alsina-Sanchís et al., 2018; Hwan Kim et al., 2020; Ola et al., 2016), which would be expected to increase both eNOS and mTOR activity downstream. We therefore next targeted Nos using L-NAME, but found no detectable effect on DA or PCV vessel size in *eng*^mu130^ embryos. Although previous evidence has suggested that eNOS may be involved in HHT due to eNOS uncoupling in *Eng* mutant endothelial cells (Toporsian et al., 2005), our data suggest that increased Nos activity in the developing zebrafish vasculature does not drive vascular malformations, at least not prior to the recruitment of vascular smooth muscle. Finally, we targeted TOR downstream of PI3K/AKT and found that rapamycin treatment normalised the enlarged vessels and reduced the excess kugel formation in *eng*^mu130^ embryos. This is consistent with previous findings in a mouse neonatal retinal model caused by loss of BMP9/10 ligands in which targeting mTOR had beneficial effects (Ruiz et al., 2020). We therefore show that independently targeting two separate pathways, MEK/ERK and Mtor, downstream of Vegf could prevent the mutant vascular phenotype in *eng*^mu130^ embryos. We then asked whether there was synergy between these two pathways by combining low-dose TOR and MEK inhibitors. At low dose, neither TOR nor MEK inhibition had any detectable effect on the developing vasculature of *eng* mutant embryos. However, when used together in combination, they efficiently reduced the vascular phenotype.

Our work signposts toward other useful avenues of research. It would be useful to examine how long abnormal phenotypes are prevented after cessation of VEGF inhibition. Histological examination of the hearts of *eng* mutants to characterise the nature of their cardiac enlargement and visualise the coronary epicardial and microvasculature would be interesting. We did attempt to perform immunostaining studies quantifying the expression and activation of Vegf and its signalling pathways in *eng* mutants with and without drug treatments, but were unable to obtain technically acceptable results due to a lack of suitable antibodies, despite attempting to reproduce other published assays. However, our findings do strongly suggest that Vegf signalling is upregulated following *eng* mutation.

Our data indicate that the HHT-like phenotype in zebrafish *eng* mutants can be mitigated through modulation of Vegf signalling and implicate synergistic targeting of ERK and mTOR pathways as a therapeutic strategy in HHT. The ability to combine subtherapeutic doses of these inhibitors might reduce the risks of toxicity while ameliorating the consequences of HHT.

## MATERIALS AND METHODS

### Reagents

All chemicals were dissolved in E3 medium and administered by immersion from 48 to 74 hpf. Vegf signalling was inhibited using 25 nM tivozanib (AV-951, AVEO Pharmaceuticals); vehicle control groups were exposed to 0.0025% dimethyl sulfoxide (DMSO). mTOR signalling was inhibited using 2-2.5 µM rapamycin (Sigma-Aldrich); vehicle control groups were exposed to 0.2-1% DMSO. MEK signalling was inhibited using 7.5-10 µM PD0325901 (Anastasaki et al., 2012) (Sigma-Aldrich); vehicle control groups were exposed to 0.75-1% DMSO. p38 MAPK signalling was inhibited by 25 µM SB 203580 (Sigma-Aldrich). Nos inhibition was achieved by incubation with 0.5 mM L-NAME (Sigma-Aldrich) diluted in E3 medium.

### Animal models

Zebrafish (*Danio rerio*) experiments were performed at the University of Sheffield under Home Office project licences PPL 70/8588 and PP3256323. The *eng* mutant (*eng*^mu130^) (Sugden et al., 2017) was kindly provided by Dr Arndt Siekmann. *eng*^mu130^ lines were raised in *Tg(kdrl:Hsa.HRAS-mCherry)^s916^* (Chi et al., 2008) and *Tg(fli1a:nEGFP)^y7^* (Roman et al., 2002) transgenic backgrounds, which label the EC membrane and endothelial nuclei, respectively*.* Adult fish were housed in a recirculating aquarium with a 14-h light/10-h dark cycle at 28.0±1°C, pH 7.5 and 80% oxygen saturation. Clutches of sibling embryos were generated by pair-mating male and female heterozygous adults to generate mixed WT, heterozygous (+/−), and homozygous (−/−) mutant offspring. Embryo age is stated with a tolerance of ±2 hpf to allow for slight differences in experimental length.

### Morphology of adult zebrafish hearts and retinae

*Tg(kdrl:Hsa.HRAS-mCherry)^s916^* adult fish (5-6 months) were culled by a lethal dose of tricaine (MS-222). Freshly dissected hearts were fixed in 4% paraformaldehyde overnight at 4°C. Hearts were weighed, and imaged using a stereomicroscope (Olympus IX81). To prepare retinae, heads were fixed in 4% paraformaldehyde overnight at 4°C. Eyes were enucleated, and retinae were dissected, flat-mounted onto glass slides, and imaged using a fluorescent stereomicroscope (Axio Zoom V.16). Number of capillary interconnections, optic artery diameter and vessel branching were quantified as previously described (Wiggenhauser et al., 2017).

### Genotyping

After experimental observations were complete, anaesthetised embryos were individually placed in microcentrifuge tubes with 25 μl 50 mM NaOH, heated to 95°C for 10 min, then cooled to 10°C. The reaction was neutralised by addition of 0.5 μl 100 mM Tris-HCl pH 9.5 (Sigma-Aldrich). Genomic DNA was amplified by PCR (MultiGene™ OptiMax Thermal Cycler) as previously described (Sugden et al., 2017) using primers (FWD, 5′-GCTGATTAGGGCTGCAAGA-3′; REV, 5′-TGTTGTGGTAATTTTACAGTTGCT-3′) to generate a 418 bp DNA fragment. Restriction digest at 37°C for 20 min with *Msp*1 (New England Biolabs) was performed to cleave the WT, but not *eng*^mu130^, PCR product into 246 bp+172 bp fragments, visualised using agarose gel electrophoresis.

### Quantification of embryonic blood vessel diameter

Zebrafish embryos aged 2-4 dpf were imaged for red and/or green fluorescence using a fluorescent stereomicroscope (Zeiss Axio Zoom V.16) then anaesthetised using 0.4% tricaine and mounted in 1% low-melting-point agarose (Biolabs) within a 1 mm diameter glass capillary. Samples were suspended in the chamber of a Zeiss Z1 light-sheet microscope filled with E3 medium at 28°C. Samples were excited with a 488 nm and 561 nm wavelength light sheet, and the emitted GFP and RFP signals were detected using an LP560 filter. Image acquisition and processing was performed using ZEN Black software (Zeiss). *Z*-stacks of the trunk and head vasculature were used to generate maximum-intensity projections. Diameters of the DA and PCV were measured using Fiji image analysis software (Schindelin et al., 2012) at the midway point between two ISVs along the yolk extension, five points in total, to generate mean diameter per embryo as previously described (Sugden et al., 2017). For ISV diameter, three measurements were made along three ISVs between the DA and DLAV to calculate average ISV diameter for both arterial and venous ISVs. Diameter of the BA was measured at three points along the vessel, and mean diameter was calculated for each embryo. Kugeln were identified and counted as previously described (Kugler et al., 2019). Cerebral vessel development was quantified using a recently described automated analysis pipeline (Kugler et al., 2022).

### Heart rate measurement

Individual non-anaesthetised embryos were observed under a bright-field stereomicroscope (Olympus IX81). Heart rate was calculated over 15 s, three times per embryo, expressed as beats/min.

### Statistical analysis

The investigator was unaware of genotype or drug treatment when performing analyses. Statistical analysis was performed in GraphPad Prism 7. Data were subjected to D'Agostino-Pearson normality test before analysis. Statistical tests used are indicated in figure legends. Error bars display s.d. Each experiment was repeated three times, unless otherwise stated. *P*<0.05 was considered significant.

## Supplementary Material

10.1242/dmm.049567_sup1 Supplementary information
