# Supplementary material for: Therapeutic targeting of vascular malformation in a zebrafish model of hereditary haemorrhagic telangiectasia
Source: Dis Model Mech. 2023 Mar 30;16(4):dmm049567. doi: 10.1242/dmm.049567 (PMC10110397; doi:10.1242/dmm.049567)
Supplement: Supplementary information [file dmm-16-049567-s1.pdf]

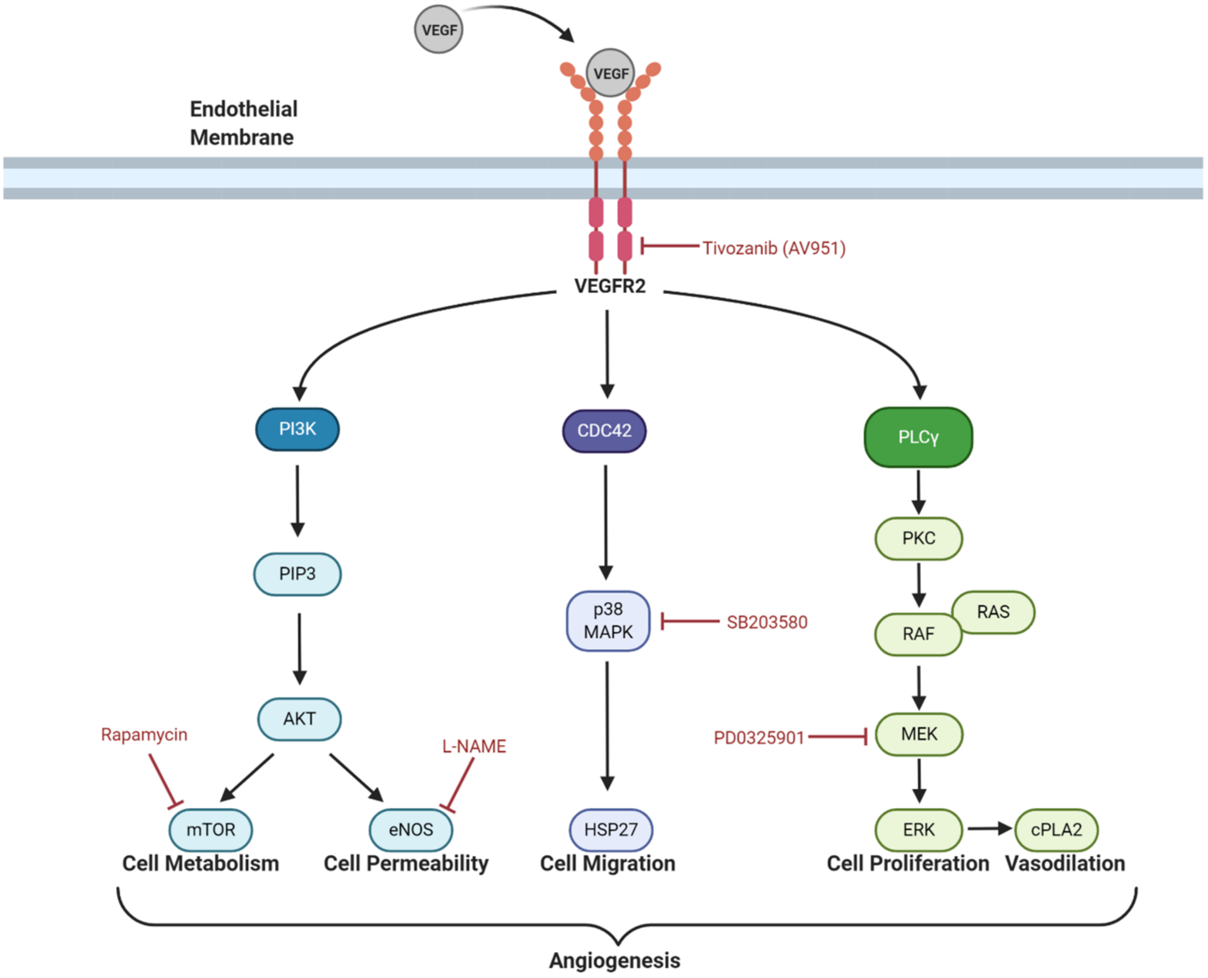

Fig. S1. Schematic diagram summarising VEGF signalling in endothelial cells. Drugs targeting VEGFR2 and enzymes in different downstream pathways are shown in red. Figure created with BioRender (https://biorender.com/). (Adapted from (Snodgrass et al., 2021)).

Disease Models & Mechanisms • Supplementary information

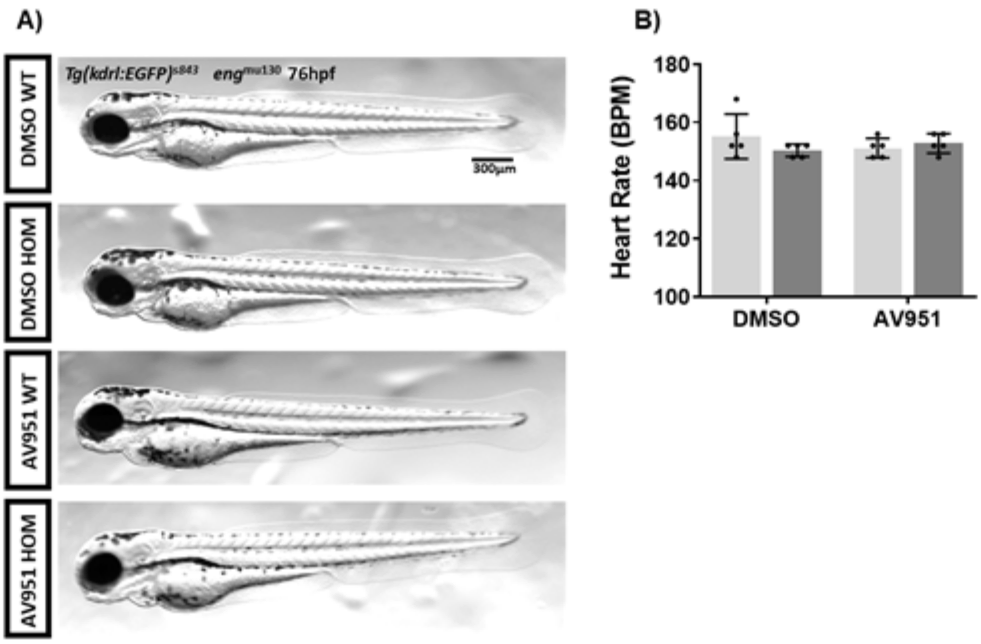

Fig. S2. WT and *eng*^{mu130} mutant embryos display no obvious morphological differences when treated with VEGF inhibitors. A) Representative images of 3dpf WT and *eng*^{mu130} homozygous (HOM) mutant embryos with and without AV951 (50nM) treatment for 24h. B) Quantification of heart rate for WT and *eng*^{mu130} mutant embryos with and without AV951 treatment. (5/group).

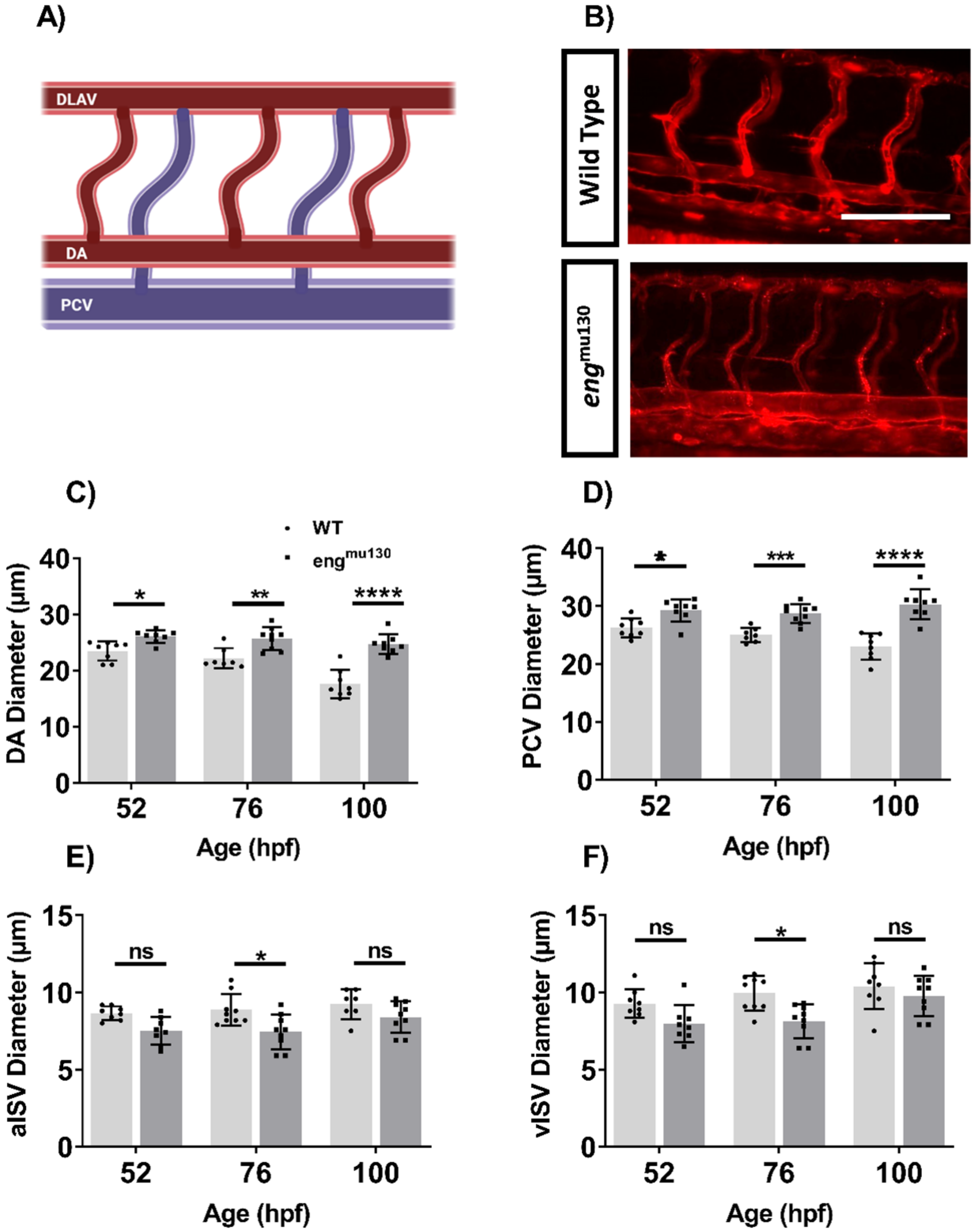
A)
DLAV
DA
PCV
B)
Wild Type
eng^{mu130}
C)
WT
eng^{mu130}
DA Diameter (µm)
Age (hpf)
D)
PCV Diameter (µm)
Age (hpf)
E)
aISV Diameter (µm)
Age (hpf)
F)
vISV Diameter (µm)
Age (hpf)

Fig. S3. *eng*^{mu130} mutant embryos display increased diameters in the dorsal aorta and cardinal vein compared with control siblings. A) Schematic diagram of the region of interest. Figure created with BioRender (https://biorender.com/). B) Representative maximum intensity projection of *Tg(kdrl:Hsa.HRAS-mCherry)*^{s916} WT and *eng*^{mu130} trunk vasculature at 76hpf. Abbreviations: DA, dorsal aorta; PCV, posterior cardinal vein; aISV, arterial intersegmental vessel; vISV, venous intersegmental vessel. Scale bar = 100µm. C,D,E and F) Significantly enlarged vessel diameters of DA and PCV in *eng*^{mu130} embryos compared with WT siblings. mCherry intensity was used to establish ISV lumen size. aISV, and vISV are thinner in *eng*^{mu130} mutants at 76h, and partially normalise by 100h. (one-way ANOVA with Tukey post-hoc test, 8-10/group.)

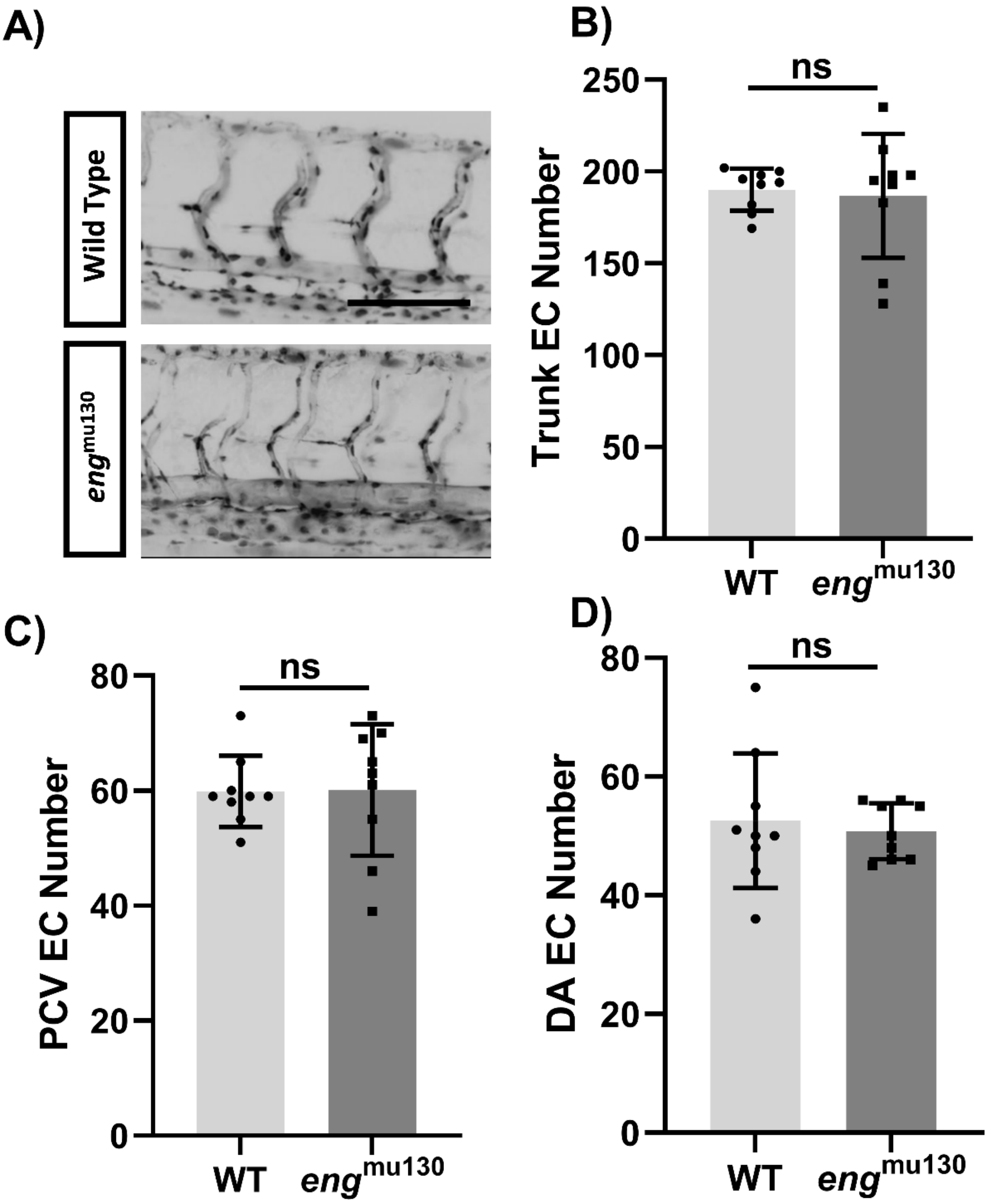

Fig. S4. *endoglin* loss of function does not affect endothelial cell numbers. A) Representative maximum intensity projection of *Tg(kdrl:Hsa.HRAS-mCherry)*^{s916}, *Tg(fli1a:nEGFP)*^{y7} trunk vasculature at 72hpf. B) Quantification of endothelial cell nuclei in all vessels in the region of interest. C) Quantification of endothelial cell nuclei in the posterior cardinal vein (PCV). D) Quantification of endothelial cell nuclei in the dorsal aorta (DA). (Unpaired student's t-test, 9/group.)

Disease Models & Mechanisms • Supplementary information

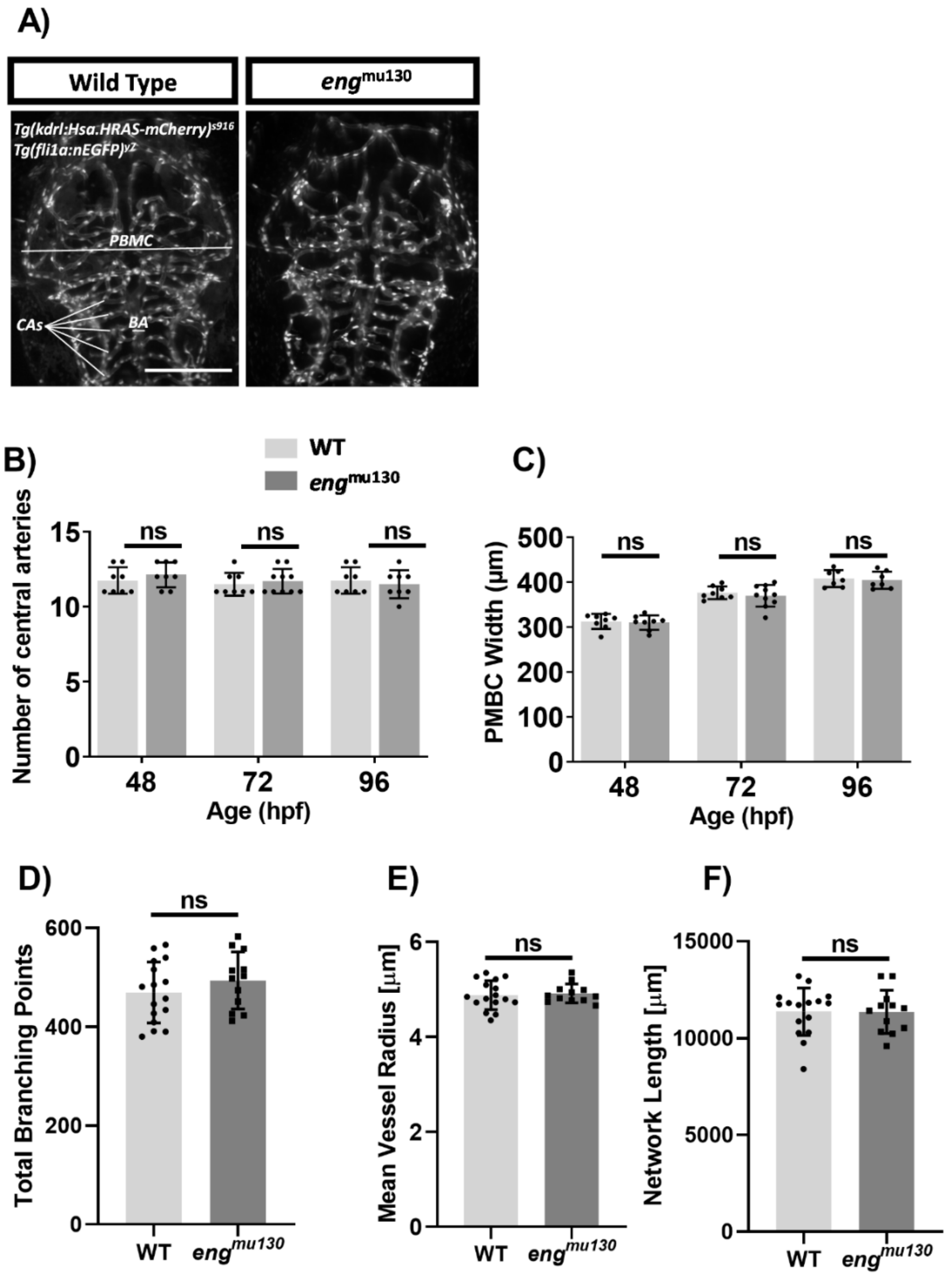
A)
Wild Type
eng mu130
Tg(kdrl:Hsa.HRAS-mCherry)s916
Tg(fli1a:nEGFP)yZ
PBMC
CAs
BA
B)
WT
eng mu130
Number of central arteries
ns
48
72
96
Age (hpf)
C)
PMBC Width (µm)
D)
Total Branching Points
E)
Mean Vessel Radius [µm]
F)
Network Length [µm]
WT
eng mu130

Fig. S5. *eng*^{mu130} mutant cranial vasculature phenotypic analysis compared with wild-types at 2-4 dpf. A) Representative maximum intensity projection of the region of interest in 3dpf embryonic cranial vasculature. *Tg(kdrl:Hsa.HRAS-mCherry)*^{s916} labels endothelial cell membrane and *Tg(fli1a:nEGFP)*^{y7} labels endothelial cell nuclei. Abbreviations: BA, basilar artery; PMBC, posterior midbrain channel; CA, central arteries. Scale bar = 150µm. B) The number of midbrain central arteries is similar in *eng*^{mu130} and wild-type siblings. Data analysed by one-way ANOVA with Tukey post-hoc test. n = 8-10/group. C) PMBC diameter is similar in *eng*^{mu130} and wild-type siblings. Data analysed by one-way ANOVA with Tukey post-hoc test. n = 8-10/group. D, E, F) An automated image analysis pipeline was used to quantify total branching points, mean vessel radius, and total network length in 72hpf wildtype and *eng*^{mu130} embryos. Data analysed by unpaired student's t-test. n = 15/group.

Disease Models & Mechanisms • Supplementary information

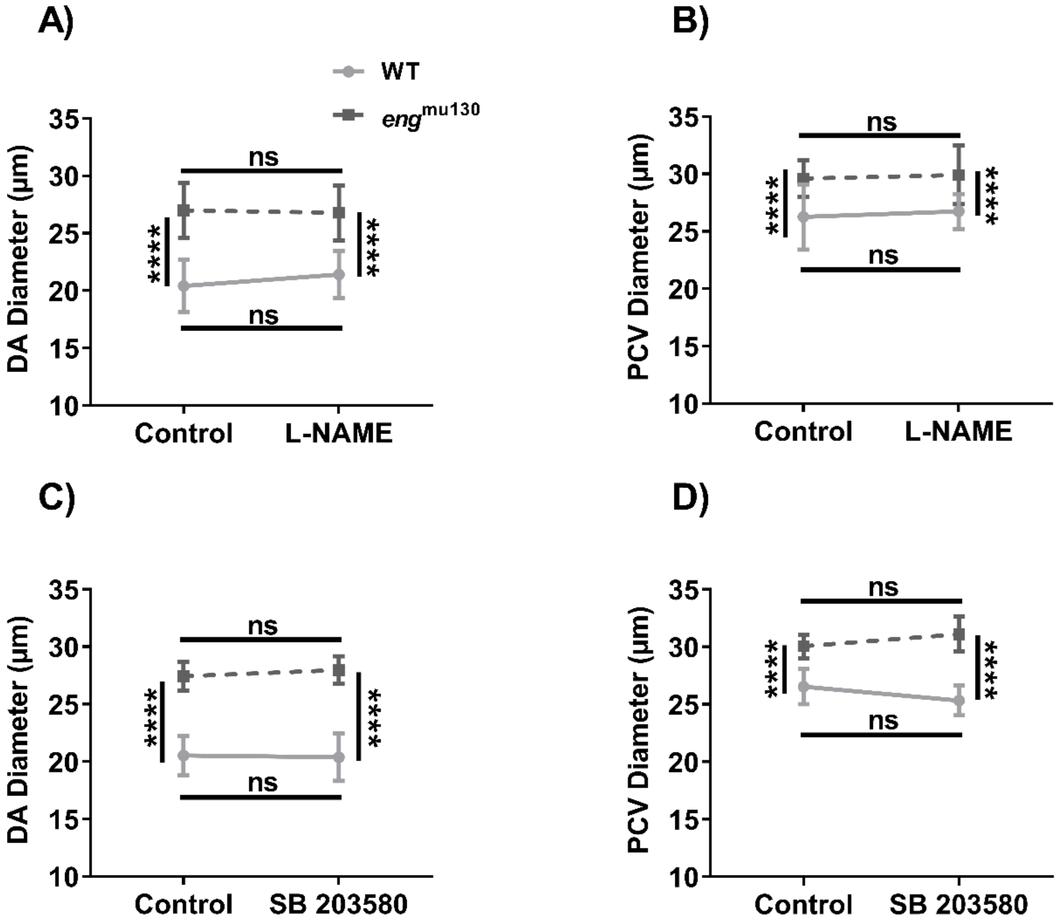

Fig. S6. NOS or p38 MAPK inhibition between 2-3dpf does not affect the phenotype of *eng*^{mu130} and WT embryos. A, B) Quantification of vessel diameter for the DA and PCV in WT and *eng*^{mu130} mutant embryos with and without L-NAME treatment. C, D) Quantification of vessel diameter for the DA and PCV in WT and *eng*^{mu130} mutant embryos with and without SB 203580 treatment. (two-way ANOVA with Tukey post-hoc test, 10-15/group.)

Disease Models & Mechanisms • Supplementary information
